# 

**Published:** 1898-03-05

**Authors:** 


**Notices of Births, Deaths, and Marriages** are inserted in this column at a charge of 2s. 6d. for an announcement not exceeding four lines.

### NOTICE TO CORRESPONDENTS.

All MS., Letters, Books for Review, and other matters intended for the Editor should be addressed The Editor, "The Hospital," The Hospital Building, 28 & 29, Southampton Street, Strand, W.C.

The Editor cannot undertake to return rejected MS., even when accompanied by stamped directed envelope.

**Notice to Advertisers and Subscribers.**

All Advertisements, Orders for Copies of The Hospital, and Business Communications should be addressed to **THE MANAGER, (Not to The Editor),** The Hospital, The Hospital Building, 28 & 29, Southampton Street, Strand, London, W.C.

The Cost of Subscription, post free, is as follows:—Per week, 2½d. per quarter, 2s. 8d.; per half-year, 5s. 3d.; per annum, 10s. 6d. Foreign:—Per annum, 15s. 2d.; payable, in all cases, in advance.

**NOTICE.—Vol. XXII. of "The Hospital" (April, 1897, to September, 1897), in handsome cloth binding, is now on sale at the Office of this Journal, price 7s. 6d. Cases for binding the Numbers contained in this Volume, 1s. 6d. Index to Vol. XXII., 2½d., post free. The Monthly Part for March is now ready. Price 6d., by post 8d.**

### SCRAPS AND GLEANINGS.

The Chelsea Hospital for Women has received £200, part of bequest of the late Richard Gibbs, Esq., as awarded by the executors.

The Worshipful Company of Leathersellers have contributed a further donation of 10 guineas to the Extension Fund of the Miller Hospital, Greenwich.

Owing to a falling off in annual subscriptions and congregational collections for some years past, the accounts of the Frome Cottage Hospital have shown a deficit. This has gone on increasing, until at the end of 1897 it amounted to £102. A sale of part of the reserve fund has been decided on in order to pay this off.

The patients treated at the dispensaries mentioned below during 1897 were as follows: Barnstaple and North Devon, 2,812, against 2,891 in 1896; Bath Eastern Dispensary, 4,340, against 4,437; Exmouth Dispensary, 944; Edinburgh Royal Dispensary, 9,450, and 2,942 visited at their own homes; Glasgow Central Dispensary, 6,932; Lincoln General, 3,863; and the Bristol Dispensary, 10,879.

The Grosvenor Hospital for Women and Children, Vincent Square, Westminster, has received a legacy of £50 (free of duty) under the will of the late Miss S. E. Aldridge, of 36, Rutland Gate, S.W.

By 25 votes to 17 the Norwich Town Council has declined for the present to sanction the expenditure which the Sanitary Committee recommended should be made in doubling the accommodation of the solation Hospital.

The present Lord Mayor of Birmingham, seeing the inequality which exists in the Hospital Sunday collections each year in that town, proposes that the triennial system under which the General Hospital receives the whole of the collection one year, Queen Hospital the whole the next, and the remaining charities of Birmingham the whole sum the next year, shall be abolished, and that in future all the institutions should benefit each year. He does not propose to alter the present system of distribution, but suggests that the General and Queen's Hospitals should still draw one-third each of the amount collected.

